# Cellularly-Driven Differences in Network Synchronization Propensity Are Differentially Modulated by Firing Frequency

**DOI:** 10.1371/journal.pcbi.1002062

**Published:** 2011-05-19

**Authors:** Christian G. Fink, Victoria Booth, Michal Zochowski

**Affiliations:** 1 Department of Physics, University of Michigan, Ann Arbor, Michigan, United States of America; 2 Departments of Mathematics and Anesthesiology, University of Michigan, Ann Arbor, Michigan, United States of America; 3 Biophysics Program, University of Michigan, Ann Arbor, Michigan, United States of America; École Normale Supérieure, College de France, CNRS, France

## Abstract

Spatiotemporal pattern formation in neuronal networks depends on the interplay between cellular and network synchronization properties. The neuronal phase response curve (PRC) is an experimentally obtainable measure that characterizes the cellular response to small perturbations, and can serve as an indicator of cellular propensity for synchronization. Two broad classes of PRCs have been identified for neurons: Type I, in which small excitatory perturbations induce only advances in firing, and Type II, in which small excitatory perturbations can induce both advances and delays in firing. Interestingly, neuronal PRCs are usually attenuated with increased spiking frequency, and Type II PRCs typically exhibit a greater attenuation of the phase delay region than of the phase advance region. We found that this phenomenon arises from an interplay between the time constants of active ionic currents and the interspike interval. As a result, excitatory networks consisting of neurons with Type I PRCs responded very differently to frequency modulation compared to excitatory networks composed of neurons with Type II PRCs. Specifically, increased frequency induced a sharp decrease in synchrony of networks of Type II neurons, while frequency increases only minimally affected synchrony in networks of Type I neurons. These results are demonstrated in networks in which both types of neurons were modeled generically with the Morris-Lecar model, as well as in networks consisting of Hodgkin-Huxley-based model cortical pyramidal cells in which simulated effects of acetylcholine changed PRC type. These results are robust to different network structures, synaptic strengths and modes of driving neuronal activity, and they indicate that Type I and Type II excitatory networks may display two distinct modes of processing information.

## Author Summary

Synchronization of the firing of neurons in the brain is related to many cognitive functions, such as recognizing faces, discriminating odors, and coordinating movement. It is therefore important to understand what properties of neuronal networks promote synchrony of neural firing. One measure that is often used to determine the contribution of individual neurons to network synchrony is called the phase response curve (PRC). PRCs describe how the timing of neuronal firing changes depending on when input, such as a synaptic signal, is received by the neuron. A characteristic of PRCs that has previously not been well understood is that they change dramatically as the neuron's firing frequency is modulated. This effect carries potential significance, since cognitive functions are often associated with specific frequencies of network activity in the brain. We showed computationally that the frequency dependence of PRCs can be explained by the relative timing of ionic membrane currents with respect to the time between spike firings. Our simulations also showed that the frequency dependence of neuronal PRCs leads to frequency-dependent changes in network synchronization that can be different for different neuron types. These results further our understanding of how synchronization is generated in the brain to support various cognitive functions.

## Introduction

Neuronal synchronization is thought to underlie spatiotemporal pattern formation in the healthy [1]–[4] and pathological brain [5]–[9]. The propensity for synchronization in a neuronal network is determined by both cellular and network properties. An important experimentally obtainable measure of cellular properties is the neuronal phase response curve (PRC) [10]. The PRC characterizes the change in spike timing of a periodically firing neuron in response to brief, weak external stimulation. PRCs have been classified into two general categories: Type I, which display only phase advances in response to excitatory stimuli, and Type II, which respond with both phase advances and delays. Type I cells exhibit relatively poor propensity for synchronization under excitatory coupling, while Type II cells synchronize better [10]–[17]. Furthermore, the PRC characteristics thought to be responsible for synchronization propensity change differentially as a function of frequency for Type I and Type II cells [18]. In this study, we explain the differential effects of frequency modulation on neuronal response properties and exploit these effects to investigate differential changes in the capacity for synchronization of excitatory networks consisting of Type I or Type II neurons.

To demonstrate the universality of the frequency-dependent effects on the neuronal PRC, we consider a reduced model neuron described by the Morris-Lecar equations [19] which can display either a Type I or Type II PRC in different parameter regimes [20]. Then, to present the effects within a physiological context, we turn to the results of a recent experimental study which showed that cholinergic modulation of cortical pyramidal neurons switches the neuronal PRC from Type II to Type I [21]. In a Hodgkin-Huxley-based cortical pyramidal neuron model, the switch in PRC type was shown to depend on a slow, low-threshold potassium current which is targeted by cholinergic modulation [22]. Using these two neuronal models, we explain the underlying cellular basis of the differential frequency effects on the PRC. We show that the relative timing of hyperpolarizing, potassium currents in relation to the model's depolarizing currents (a calcium current in the Morris-Lecar model and a sodium current in the cortical pyramidal cell model) plays a crucial role in shaping the phase response of a neuron. We then investigate the influence of the frequency-dependent cellular effects on network activity by analyzing network synchronization as a function of underlying neuronal spike frequency near firing threshold in large-scale, excitatory networks composed of either Morris-Lecar neurons or cortical pyramidal model neurons. As expected, the neuronal PRC type profoundly affects network propensity for synchronization [17]. We show that, in general, increasing firing frequency near firing threshold has little effect upon synchrony in Type I networks, while it severely suppresses synchrony in Type II networks. We show these results to be robust to neuronal heterogeneity, network connectivity parameters and whether neuronal activity is driven by constant or stochastic inputs.

Our results provide important insight into differential changes in the propensity for network synchronization induced by the external modulation of neuronal frequency. As neuronal firing frequency changes, the changes in network spatiotemporal patterns depend upon the response characteristics of the individual cells in the network.

## Methods

### Morris-Lecar neuron model

We used the Morris-Lecar model [19] as a generic neuronal model to initially explore frequency-dependent PRC effects. The model contains two active ionic currents: an inward 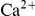 current whose dynamics are instantaneous and an outward 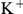 current gated by the dynamic variable 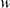. The current balance equation for the 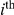 cell is
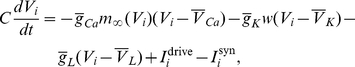
(1)where 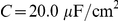, 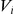 is in millivolts, 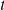 is in milliseconds, 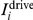 is an externally applied current measured in 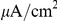, and 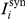 is the synaptic current received by neuron 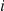. The 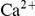 current is governed by the steady-state activation function 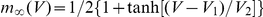, while dynamics of the 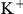 current gating variable 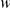 are given by 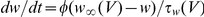, with 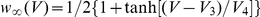 and 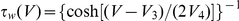.

The Type I and Type II neuronal models share the parameter values 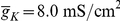, 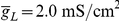, 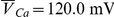, 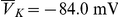, 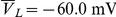, 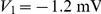, and 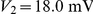. Type I cells are modeled with 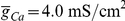, 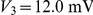, 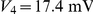, and 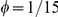, while Type II cells are modeled with 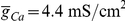, 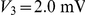, 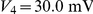, and 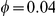. These values were taken from [20].

### Cortical pyramidal neuron model with simulated acetylcholine modulation

The cortical pyramidal model neuron we employed was motivated by recent computational and experimental findings, as reported in [22]. Varying the maximum conductance of a 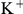-mediated adaptation current, 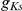, from 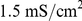 to 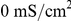 effectively switches the response characteristics of the cortical pyramidal model neuron from Type II to Type I, a phenomenon which has been observed *in situ* and simulates the effects of cholinergic neuromodulation [21]. The model also features a fast, inward 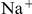 current, a delayed rectifier 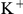 current, and a leakage current, in addition to the aforementioned slow, low-threshold 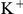 current responsible for spike-frequency adaptation [22], [23]. The current balance equation for the 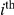 cell is
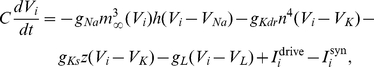
(2)


with 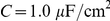, 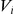 in millivolts, and 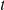 in milliseconds. 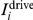 is an externally applied current measured in 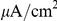, and 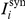 is the synaptic current received by neuron 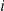.

Activation of the 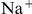 current is instantaneous and governed by the steady-state activation function 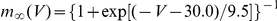. Dynamics of the 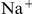 current inactivation gating variable 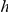 are given by
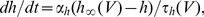
(3)


with 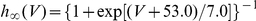 and 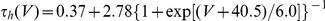. The delayed rectifier 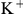 current is gated by 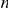, whose dynamics are governed by
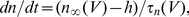
(4)


with 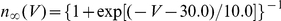 and 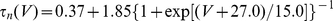. The slow, low-threshold 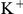 current targeted by cholinergic modulation is gated by 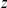, which varies in time according to
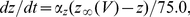
(5)


where 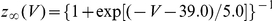. The parameters 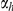 and 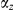 in the current gating equations are varied in the investigation of the underlying cellular basis of the differential frequency effects on the PRC, but they are set to 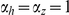 in the network simulations.

**Figure 1:**
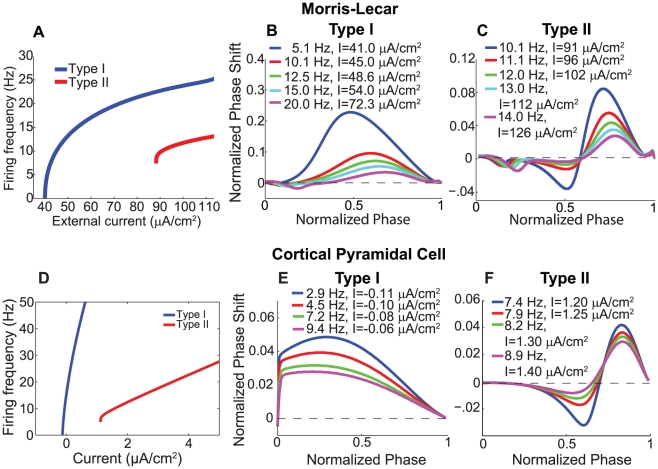
Neuronal response characteristics of Type I and Type II neurons for Morris-Lecar and cortical pyramidal cell models. (A) Frequency-current curve for Type I and Type II Morris-Lecar model neurons. Note that the Type I cell can fire at arbitrarily low frequencies, while the Type II cell exhibits a non-zero frequency threshold. (B,C) Frequency dependence of PRCs for Morris-Lecar model neurons with Type I and Type II response characteristics. When the PRC was computed at different neuronal firing frequencies (different curves), amplitudes of phase shifts were attenuated, and the Type II neuron showed asymmetric attenuation of the phase advance and phase delay regions. (D) Frequency-current curves for Type I (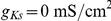, cholinergic modulation) and Type II (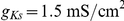, no cholinergic modulation) cortical pyramidal model neurons. The Type I neuron could fire at arbitrarily low frequencies, while the Type II neuron exhibited a threshold frequency of approximately 8 Hz. (E) PRCs for different firing frequencies of the Type I cortical pyramidal neuron. (F) PRCs for different firing frequencies of the Type II cortical pyramidal neuron. In both models, the Type I cells exhibited global attenuation of the phase responses, while increased firing frequency evoked asymmetric attenuation in the phase delay region as compared to the phase advance region in Type II cells.

The slow, low-threshold 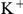 current loosely models the muscarine-sensitive M-current observed in cortical neurons. It has been shown *in silico* that eliminating this current is sufficient to switch the model neuron's PRC from Type II to Type I [22]. This is intended to model cholinergic neuromodulation, which has been shown experimentally to switch cortical pyramidal neurons between Type I and Type II phase responses [21]. This switching of PRC profile is demonstrated in Fig. 1, and is obtained by setting 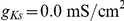 to obtain a Type I response (simulated cholinergic modulation) and 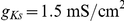 to obtain a Type II response (simulated absence of cholinergic modulation). All other parameter values are the same for both types of neurons: 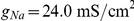, 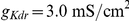, 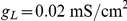, 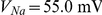, 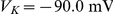, and 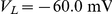.

### PRC calculation

For both neuronal models, 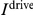 is set to a fixed value to elicit repetitive firing in a single, synaptically isolated neuron, and the model equations are time evolved using a fourth-order Runge-Kutta numerical scheme until the oscillatory period stabilizes. Then, using initial conditions associated with spike peak, brief current pulses are administered at different phases of the oscillation, and the perturbed periods are used to calculate the corresponding phase shifts. The current pulses are administered at 100 equally-spaced time points throughout the period of the neuronal oscillation. The current pulses have a duration of 0.06 ms and an amplitude of 3.0 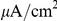 for the Type I cortical pyramidal neuron, a duration of 0.06 ms and an amplitude of 10.0 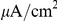 for the Type II cortical pyramidal neuron, and a duration of 0.50 ms and an amplitude of 100.0 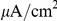 for both the Type I and Type II Morris-Lecar neurons.

### Network simulations

In all network simulations, the number of neurons is 200, and the synapses are exclusively excitatory. The network connectivity pattern is constructed using the Watts-Strogatz architecture for Small World Networks [24]. Starting with a 1-D ring network with periodic boundary conditions, each neuron is at first directionally coupled to its 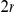 nearest neighbors, and then every connection in the network is rewired with probability 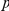 to another neuron selected at random. In this way, 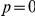 results in a locally-connected network and 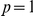 in a randomly connected network. The radius of connectivity 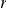 therefore determines the density of connections in the network, while the re-wiring parameter 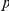 determines the network connectivity structure. Network connectivity 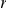 is set to 4 in all simulations.

Synaptic current is transmitted from neuron 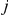 at times 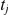 when its membrane voltage breaches −20 mV. The synaptic current delivered from neuron 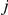 to a synaptically connected neuron 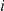 at times 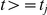 is given by 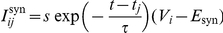. The total synaptic current to a neuron 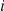 is simply given by 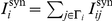, where 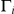 is the set of all neurons which synapse onto neuron 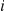. The synaptic weight *s* is the same for all synapses within a given simulation, and we set 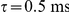 and 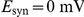. All simulations are run for 10,000 ms, with the first 3000 ms disregarded in order to eliminate initial transient effects. The dynamics are numerically integrated in Matlab using a fourth-order Runge-Kutta method with a time step of 0.05 ms for the cortical pyramidal neuron networks and 0.10 ms for Morris-Lecar neuron networks.

We employ two different methods to modulate network firing frequency in our simulations. The first is to simply modulate the supra-threshold value of 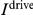 for all neurons in the network. In order to prevent the networks from trivially synchronizing, we do not supply each neuron with exactly the same level of current, but instead sample from a Gaussian distribution of current values. The mean value of the distribution determines the average firing frequency of the network, and the standard deviation of the Gaussian is chosen such that the standard deviation in natural neuronal frequencies is 1 Hz.

In order to model more biologically relevant environmental inputs, we also run simulations of cortical pyramidal neuronal networks in which frequency is modulated by stochastic input. All neurons are given the same constant sub-threshold baseline current, plus square current pulses randomly delivered to each neuron at a specified frequency 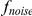, so that 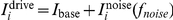. The delivery of the square current pulses is a Poisson process. Modulation of this noise frequency thereby modulates the average frequency of the network. In our simulations of stochastically-driven cortical pyramidal neuronal networks, 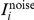 consists of square current pulses with amplitude 30 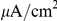 and duration 0.2 msec. With these values, at least two successive pulses are required to elicit neuronal firing. The baseline currents are 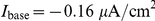 for Type I networks and 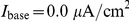 for Type II networks.

We monitor phase-synchronization of neuronal firing in our simulations using the mean phase coherence (MPC) measure, 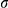 [25]. This measure quantifies the degree of phase locking between neurons, assuming a value of 0 for completely random spiking and 1 for complete phase locking. Note that MPC may be attained for locking of phases at *any value*, not just zero. The MPC between a pair of neurons, 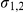, is defined by:
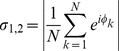
(6)

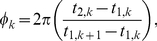
(7)


where 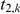 is the time of the 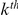 spike of neuron 2, 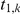 is the time of the spike of neuron 1 that is largest while being less than 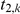, 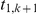 is the time of the spike of neuron 1 that is smallest while being greater than or equal to 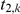, and 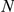 is the number of spikes of neuron 2. The MPC of the entire network, 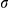, is calculated by averaging 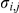 over all pairs of neurons, excluding 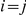. Note that this measure is not symmetric.

We quantify phase-zero synchronization of a network by calculating the bursting measure *B*, which is 0 for random spiking and approaches 1 for perfect locking at phase zero between all neurons, for a large number of total spikes and neurons. Calculation of *B* requires a time-ordered list of the spike times of all neurons over the duration of the entire simulation [26]. Denoting as 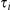 the time difference between spikes *i* and *i+1*, which do not necessarily (and probably do not) correspond to spikes of the same neuron, *B* is then defined as
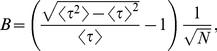
(8)


where 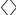 represents averaging over all spikes. This measure makes use of the fact that an ensemble of spike time intervals will have a larger standard deviation in a synchronous signal than in an asynchronous signal. In our simulations, both the mean phase coherence and the bursting parameter are calculated for neuronal activity from 3000 ms to 10,000 ms, unless otherwise noted.

## Results

We first investigate the underlying cellular basis of the differential frequency effects on Type I and Type II PRCs. We show that the relative activation levels and timing of hyperpolarizing, potassium currents in relation to depolarizing currents play a crucial role in shaping the phase response of a neuron. We then show that individual neuronal spiking frequency modulates network synchrony in significantly different ways for networks consisting of Type I or Type II cells. Specifically, synchrony in Type I networks is affected very little by frequency modulation near threshold, whereas in Type II networks, synchrony falls dramatically as frequency increases above firing threshold. This effect is due to the disparity in the frequency-modulated attenuation of the PRCs of the two cell types. We first show this effect in excitatory networks composed of Morris-Lecar model neurons, and then investigate it in depth for excitatory networks consisting of model cortical pyramidal cells under acetylcholine modulation.

### Frequency modulation of neuronal phase responses

Fig. 1 displays the response properties of the model neurons in our simulations, with Fig. 1A,D showing the frequency-current curves of the model neurons and Fig. 1B,C,E,F showing the PRCs of the model neurons. Type I PRCs in both the Morris-Lecar and the cortical pyramidal neuron models exclusively display phase advances (positive PRC values) in response to excitatory perturbations (Fig. 1B,E) while Type II PRCs show phase delays (negative PRC values) at earlier phases and advances at later phases (Fig. 1C,F). (Note that the presence of small negative regions early in Morris-Lecar PRCs and the absence of such regions in cortical pyramidal cell PRCs is a consequence of the fact that spikes consume a much larger portion of the interspike interval in the Morris-Lecar model than in the cortical pyramidal cell model [20]. We therefore ignore these early regions in Morris-Lecar PRCs.) The switch from Type I to II is induced by changes in the steady state activation function of the 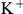 current in the Morris-Lecar model and by the presence of the slow, low-threshold 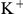 current in the cortical pyramidal cell model. A categorization of Type I and Type II can also be applied to a neuron's frequency-current (f-I) relation, with Type I f-I curves exhibiting arbitrarily low frequencies at firing thresholds and Type II f-I curves showing a finite, non-zero firing frequency at threshold. While the categorization of a neuron's PRC and f-I curve are not necessarily the same, and the relationship between the curves has not been completely determined [27], for both models considered here, PRC and f-I curve types coincide (Fig. 1A,D).

In both models, increasing frequency by increasing the constant applied current results in an attenutation of phase responses (Fig. 1). This attenuation occurred in qualitatively different ways for Type I and Type II neurons. In the Type I model neurons, increased firing frequency led to diminished phase advances but did not change the relative shape of the curves–they all remained distinctly Type I (Fig. 1B,E). In the Type II model neurons, however, there was much greater attenuation of the phase-delay region compared to the phase-advance region (Fig. 1C,F). This asymmetric attenuation can affect synchronization properties because the phase-delay region contributes to the increased propensity for synchronization in Type II excitatory networks [12]. Previously, the emergence of phase delay regions at low firing frequencies was attributed to decreased activation of 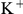-mediated adaptation currents at low frequencies [12], [18], but this explanation cannot apply to the Morris-Lecar model, since it contains no adaptation currents. Below we discuss the properties of a cell's hyperpolarizing and depolarizing currents that are responsible for its phase response, and which explain the observed frequency-dependent attenuation.

In both models, phase delays exist in the Type II parameter regimes because there is a voltage interval in which activation of an outward, hyperpolarizing current is greater than activation of the inward, depolarizing current. In the Type II Morris-Lecar model, the steady state activation curve of the 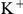 current, 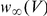, is shifted to the left and steeper compared to that of the 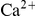 current, 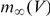, thus providing for this voltage interval. In the Type II cortical pyramidal neuron model, the steady state activation curve, 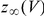, of the slow, low-threshold 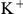 current (which is absent in the Type I neuron), is similarly shifted to the left relative to the steady-state activation curve of the 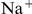 current, 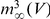. In either model, as the voltage trajectory passes through the early part of the interspike interval, a brief, excitatory stimulus will induce a larger response from the lower-threshold 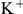 current than from the inward current, resulting in negative values of the PRC at early phases. At higher voltage levels later in the interspike interval, the inward current dominates the response to the brief stimulus due to its faster (instantaneous) activation dynamics, thus leading to advances in the cycle, and positive values of the PRC at later phases.

As firing frequency increases, the cycle trajectory passes through this 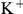-dominant voltage interval at a faster rate, thus preventing the full 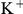 response from developing before reaching voltage levels where the instantaneous inward current can respond. The delaying 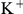 response to the brief stimulus is thus diluted by the advance-promoting inward current response, and phase delays are attenuated. This attenuation of phase delays is therefore the result of a disparity between the *fixed* dynamics of the delay-inducing 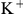 current and the time afforded that current to act by the *shrinking* interspike interval. Phase advances are less sensitive to frequency modulation since the instantaneous dynamics of the inward currents in both models can directly track the faster cycle trajectory.

**Figure 2:**
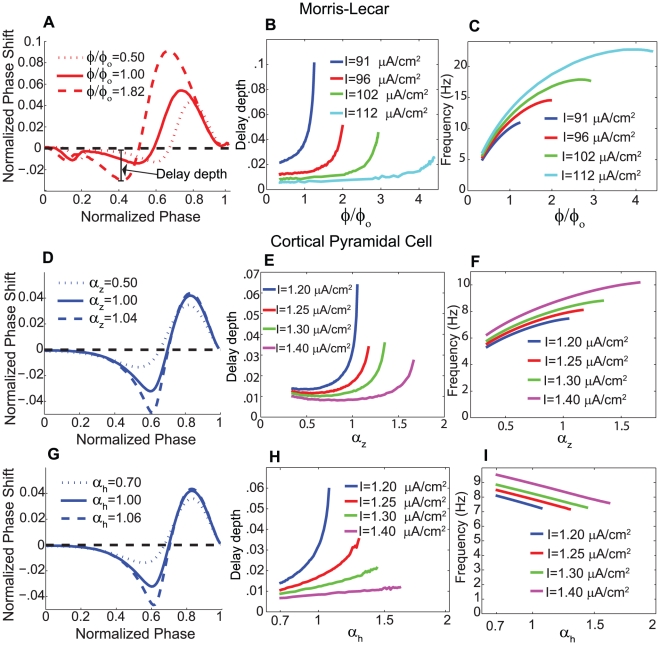
Effects of modifying speed of intracellular currents upon depth of PRC delay in Type II neurons. (A–C) Effects of modifying the speed of the potassium current in the Type II Morris-Lecar neuron, with increasing values of 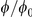 implying faster dynamics (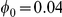). (A) PRCs of the neuron for three sample values of 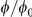, with 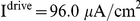. As the speed of the potassium dynamics increases, the PRC delay depths grow progressively larger. (B) Absolute value of the delay depth of the PRCs as a function of 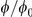, for four different values of 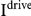, which correspond to those in Fig. 1C. (C) Neuronal firing frequency as a function of 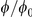, for the same values of 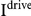 as in panel B. Note how linear growth of 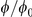 results in sub-linear growth of the frequency, indicating that the delay depth is largely determined by the speed of the potassium current relative to the spiking frequency of the neuron. (D–F) Effects of modifying the speed of the slow potassium gating variable 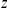 in the Type II cortical pyramidal cell model. (D) PRCs of the neuron for three sample values of 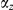, with 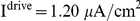. (E) Absolute value of the delay depth of the PRCs as a function of 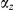, for four different values of 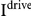, which correspond to those in Fig. 1F. (F) Neuronal firing frequency as a function of 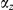, for the same values of 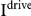 as in panel E. (G–I) Effects of modifying the speed of the sodium inactivation gating variable 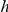 in the cortical pyramidal cell model. (G) PRCs of the neuron for three sample values of 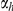, with 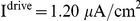. (H) Absolute value of the delay depth of the PRCs as a function of 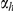, for four different values of 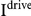, which correspond to those in Fig. 1F. (I) Neuronal firing frequency as a function of 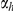, for the same values of 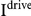 as in panel H.

We further illustrate this point by modulating the speed of the gating variable controlling the delay-inducing potassium current in each model. Fig. 2A demonstrates that in the Morris-Lecar model, increasing 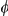, which increases the rate of the 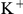 gating variable 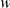, results in an increase in the amplitude of PRC phase delays, while decreasing 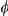 has the opposite effect. Faster 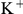 dynamics allow for faster development of the delaying 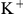 response to the excitatory stimulus. In this model, modulating 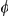 also changes the voltage levels during the interspike interval, which can shift the 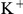 dominant voltage interval to different phases. We systematically quantify the contribution of 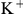 dynamics to the generation of the phase delay by measuring the changes in the PRC delay depth as a function of 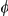 for neurons receiving different driving currents and thus exhibiting different intrinsic frequencies (Fig. 2B). The depth of the PRC delay region increased with increasing 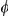 for all levels of external current, and faster-firing neurons could display similar delay depths as slower-firing neurons with appropriate increases in 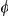. While increasing 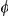 also acted to increase firing frequency (Fig. 2C), phase delay amplitudes nonetheless increased, indicating that speeding up the rate of 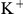 dynamics exerts a stronger effect on the phase delay than does the accompanying frequency increase.

A similar dependence of phase delay amplitude on the rate of the gating variable 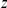 for the slow, low-threshold 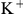 current in the cortical pyramidal cell model is shown in Fig. 2D,E. As the rate of 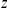 dynamics increased (i.e., as 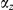 increased in Eq. 5), depths of the PRC delay increased due to the ability of the 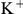 current to develop a delaying response before voltage levels were reached where the 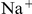 current activated. Again, increasing the rate of 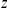 dynamics caused an increase in frequency (Fig. 2F), but the faster development of the 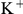 response to the perturbation could overcome a frequency-induced attenuation of phase delays. In this model, voltage levels during the interspike interval also changed with the changes in 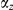, but they did not greatly influence the phase of maximal delays.

In the cortical pyramidal neuron model, the amplitude of phase delays also depended on the rate of the 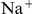 current inactivation (Fig. 2G–I), gated by the variable 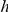 in Eq. 2. Slower 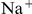 inactivation, induced by lower values of 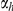 in Eq. 3, allowed larger 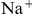 responses to the perturbing stimulus, which diluted the delaying effect of the 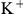 response and therefore attenuated phase delays. The rate of 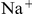 inactivation had little effect on voltage levels as a function of phase during the interspike interval, and only slightly affected the frequency. Increasing the rate of 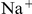 inactivation did induce a decrease in firing frequency, which would promote the observed increase in delay depth, but these changes to firing frequency were too slight to be the primary cause of the enlarged delay amplitude.

**Figure 3:**
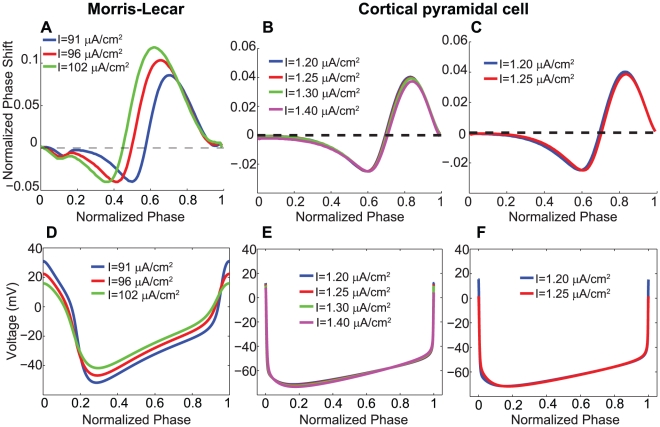
Type II PRC profiles with the same delay depth for different levels of external current. (A) PRC profiles of the Type II Morris-Lecar neuron for three different values of 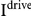, with 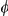 separately adjusted to induce a maximum phase delay of 0.04. (B) PRC profiles of the Type II cortical pyramidal neuron for four different values of 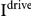, with 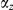 separately adjusted to induce a maximum phase delay of 0.025. (C) PRC profiles of the Type II cortical pyramidal neuron for two different values of 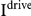, with 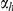 separately adjusted to induce a maximum phase delay of 0.025. (D) Unperturbed voltage traces as a function of oscillatory phase corresponding to the Type II Morris-Lecar PRCs in panel A. (E) Unperturbed voltage traces as a function of oscillatory phase corresponding to the Type II cortical pyramidal PRCs in panel B. (F) Unperturbed voltage traces as a function of oscillatory phase corresponding to the Type II cortical pyramidal PRCs in panel C. Note how the voltage traces are virtually identical in for the cortical pyramidal model, but not for the Morris-Lecar model. This explains why the PRCs are virtually identical for the cortical pyramidal model, but not the Morris-Lecar model.

These results imply that appropriate selection of the rate of variables gating the intracellular currents mentioned above permits the recovery of specified PRC delay depths for different levels of external current. Fig. 3 illustrates this effect for both models. From the curves in Fig. 2B,E,H, appropriate rates of the gating variables were separately selected for each level of external current to induce delay depths of 0.04 in the Morris-Lecar neuron and 0.025 in the cortical pyramidal neuron. In the Morris-Lecar model, the maximal phase delay region was shifted to the left as the external current increased because the voltage trace was similarly shifted (Fig. 3A,D). However, in the cortical pyramidal cell model, the PRC profiles were virtually identical for different levels of external current, both when the slow potassium current was modified and when the sodium inactivation was modified (Fig. 3B,C). This was due to the fact that the voltage traces (plotted as a function of oscillatory phase) were not shifted when either of these intracellular currents were altered (Fig. 3E,F). The invariance of the voltage traces in the cortical pyramidal cell model is an interesting phenomenon, but it is beyond the scope of this paper.

### Network correlates of PRC modulation

#### Morris-Lecar neuron network driven by constant applied currents

**Figure 4:**
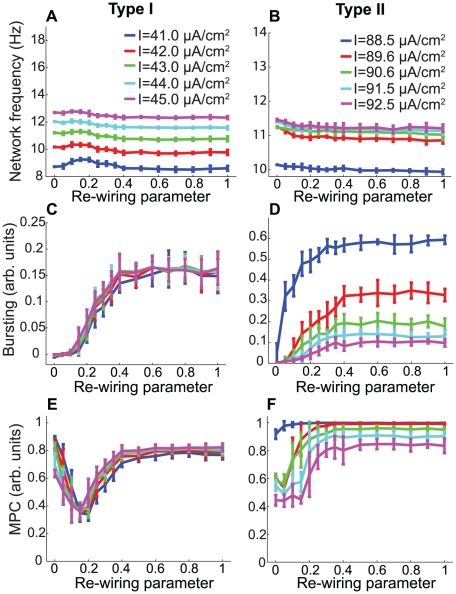
Differential effects of frequency modulation on Morris-Lecar network synchronization. Measures of network activity for simulations of large-scale (N = 200) excitatory networks of Morris-Lecar model neurons driven with various constant applied currents (different curves) for Type I (A,C,E) and Type II (B,D,F) cells. The synaptic coupling was set to 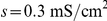 for Types I and II. (A,B) Average network firing frequency as a function of the network re-wiring parameter. (C,D) Phase-zero synchronization (as quantified by the bursting measure) versus the re-wiring parameter. (E,F) Phase locking (as measured by mean phase coherence) as a function of the re-wiring parameter.

We analyzed network activity patterns in large-scale (N = 200) excitatory networks composed of Morris-Lecar model neurons with Type I and Type II PRCs under different network connectivity regimes. As described in the Methods section, randomness of network connectivity was determined by the small-world “re-wiring parameter.” Network activity was modulated by altering the mean applied current given to each neuron, and neuronal heterogeneity was enforced by selecting applied current values from a Gaussian distribution centered on the specified mean. Fig. 4A,B show that increased mean applied current generally led to increased network frequency. Effects of the frequency-dependence of PRCs upon network synchrony are evident in Fig. 4C,D, which plot phase-zero synchronization, as measured by the bursting measure *B*, versus the re-wiring parameter for different network frequencies. In Type I networks, increased neuronal firing frequency had little effect upon synchronization, while synchronization of Type II networks substantially *decreased* with increased neuronal firing rates. Fig. 4E,F show that phase locking of the networks, as measured by mean phase coherence (MPC), painted a similar picture. For a given value of the re-wiring parameter, increased frequency had very different effects upon Type II networks in comparison to Type I networks. In fact, for 0.2≲p≲0.4, Type I network MPC discernibly increased with increased frequency, showing exactly the opposite trend as Type II networks.

#### Cortical pyramidal neuron network driven by constant applied currents

**Figure 5:**
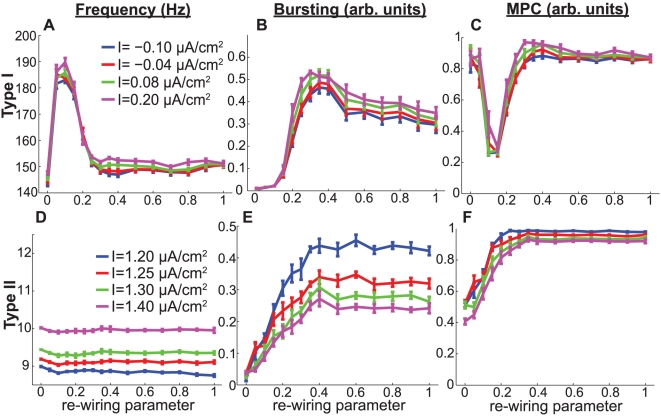
Differential effects of frequency modulation on network frequency and synchronization of cortical pyramidal cells. (A–F) Measures of network activity for simulations of large-scale (N = 200) excitatory networks of cortical pyramidal model neurons driven with varying constant applied currents for Type I (A,B,C) and Type II (D,E,F) cells. Synaptic weight was fixed at 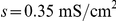 for Type I plots and 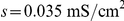 for Type II plots. (A,D) Average network frequency as a function of the re-wiring parameter for Types I and II networks. (B,E) Phase-zero synchronization, as measured by the bursting parameter, as a function of the re-wiring parameter for Types I and II networks. (C,F) Phase locking, as measured by mean phase coherence, as a function of the re-wiring parameter for Types I and II networks. Note how Type II network synchrony tended to decrease with increasing stimulation intensity, while Type I network synchrony tended to remain the same or slightly increase with increased stimulation intensity.

We first investigated synchronization properties of networks driven with constant applied currents, as in the Morris-Lecar network simulations. Every cell was driven with a constant current, 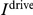, whose value was chosen from a Gaussian distribution with specified mean. This mode of driving neuronal activity reflected the conditions under which the PRC is generally computed. Fig. 5A,D show that increasing the mean value of 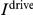 typically led to an increase in the average network firing frequency, as expected.

We observed sharp differences between responses of the Type I and Type II networks to frequency modulation. As shown in Fig. 5B, the bursting parameter tended to increase only slightly with increased applied current in Type I networks, while Fig. 5E shows that in Type II networks the phase-zero synchronization substantially decreased as applied current increased. This same trend was seen in the phase locking of the networks, albeit to a lesser degree, as shown in Fig. 5C,F. The large drop in MPC shown in Fig. 5C was due to the disruption of propagating waves as long-range connections were introduced into the network. This drop in synchrony in turn explains the large increase in frequency over the same range of the re-wiring parameter (Fig. 5A), since each neuron then receives a steady barrage of input, rather than punctuated bursts of input.

**Figure 6:**
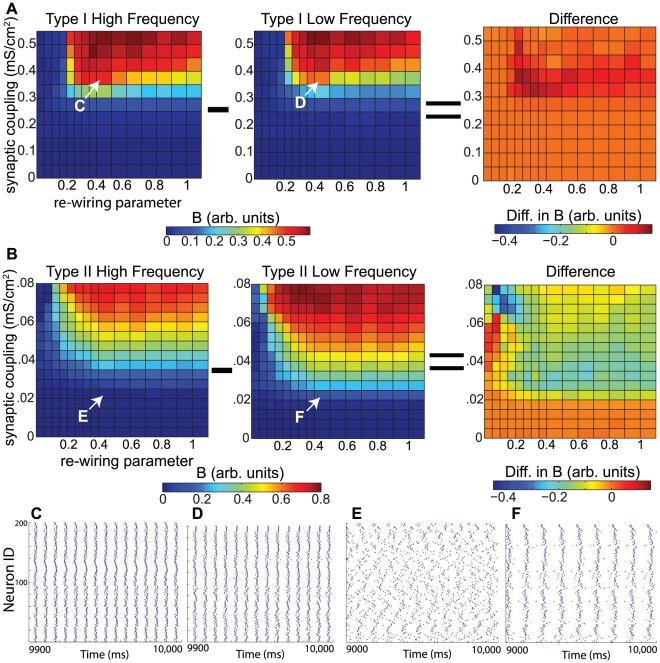
Differential effects of frequency modulation upon phase-zero synchronization in Types I and II cortical pyramidal cell networks. (A,B) Phase-zero synchrony (as measured by the bursting parameter, *B*) of Type I and Type II cortical pyramidal neuronal networks as a function of synaptic coupling strength 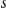 and the re-wiring parameter, 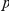. The left panels show values of *B* for networks stimulated with a high applied current (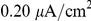 for Type I and 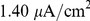 for Type II), and the middle panels show values of *B* for networks with a low applied current (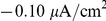 for Type I and 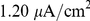 for Type II). The right panel subtracts the low-frequency values of *B* from the high-frequency values of *B*. Note the pronounced negative-difference region in the Type II plot, while the Type I plot shows almost exclusively zero or positive values of the difference. (C,D) Raster plots of the last 100 ms of simulations of high-frequency (C) and low-frequency (D) Type I networks with network parameters 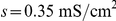 and 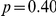. (E,F) Raster plots of the last 1000 ms of simulation of (E) high-frequency and (F) low-frequency Type II networks with network parameters 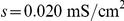 and 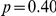. The difference in synaptic coupling values between Type I and Type II networks was due to the fact that the Type II networks synchronized better than the Type I networks and therefore required much smaller synaptic coupling values to appreciably synchronize.

To show that these results were robust to network structure and coupling strength, Fig. 6 displays how the bursting measure *B* varied with the re-wiring parameter and synaptic weight in both types of networks. Note that synaptic weights were much higher in Type I than in Type II networks because Type I networks required greater coupling in order to reach appreciable levels of synchronization. The left panels in Fig. 6A,B show the values of the bursting parameter corresponding to high-frequency networks (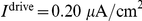 for Type I and 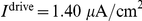 for Type II), while the center panels show the data corresponding to low-frequency networks (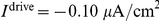 for Type I and 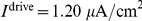 for Type II). The right panels show the difference in *B* between the high- and low-frequency networks for each network type, revealing the fundamental difference in synchronization response of the two types of networks. The right panel in Fig. 6A shows values very near zero for most of the parameter landscape, with a few slightly positive values sprinkled throughout, indicating that Type I network synchrony was largely unaffected by increased frequency, and that when increasing frequency did have an effect, it generally increased synchrony. In Type II networks, on the other hand, differences in bursting values were negative for values of *s* greater than approximately 
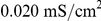
 and values of the re-wiring parameter greater than approximately 0.10. These values correspond to the parameter space in which appreciable synchronization occurred and in which propagating waves were precluded, indicating that Type II networks synchronized much better at lower frequencies for non-trivial network parameters. For many Type II network parameters, the difference in synchronization between high- and low-frequency networks was evident from the activity patterns alone (see Fig. 6E,F), while differences in Type I network synchrony were not so obvious (as in Fig. 6C,D).

#### Time to synchronization

**Figure 7:**
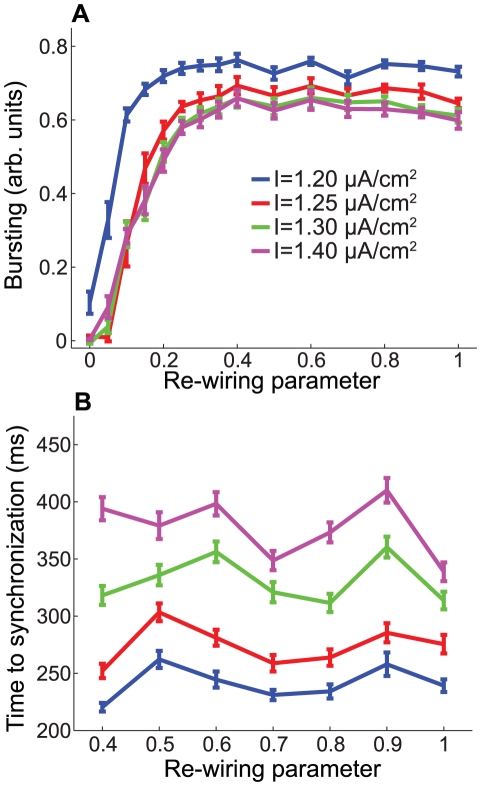
Time to synchronization for differentially-driven Type II cortical pyramidal cell networks. (A) Bursting parameter *B* as a function of the re-wiring parameter for four Type II networks driven with different values of applied current (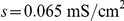). Note how for values of the re-wiring parameter greater than approximately 0.40, there was little difference among the values of *B* for different values of 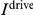, especially for the three largest values of 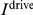. (B) Average time taken for the bursting parameter of Type II networks with randomly-distributed initial conditions to breach 0.6. Initial conditions were randomized such that initial membrane voltage values were uniformly distributed on the interval [−70 mV, −50 mV], with gating variables set to corresponding equilibrium values. Each data point is an average of 100 simulations. Note that panel B plots the subset of values of the re-wiring parameter from panel A for which the bursting parameter assumes approximately constant values.

In the high-coupling regime, where differences in steady-state synchronization between high- and low-frequency Type II networks were diminished, we investigated whether frequency might still affect the *time* to synchronization. In the right panel of Fig. 6B, it is clear that the magnitude of the difference between high- and low-frequency values of *B* in Type II networks decreased above approximately 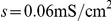. This was a saturation effect; regardless of the level of applied current, there was a ceiling of 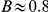 which was not breached, and as the differentially-driven networks approached this limit, the differences in their steady-state values of *B* diminished. This effect is displayed in the tightly-packed values of *B* shown in Fig. 7A. Despite this fact, our simulations showed that in this regime there was still a major difference between the differentially-driven networks: the time to synchronization. As Fig. 7B shows, when started with random initial conditions, Type II networks synchronized more quickly when driven with lower levels of applied current, even when there was very little difference between the levels of steady-state synchrony. This further underscores the enhanced synchronization properties that Type II networks exhibited at lower frequency.

**Figure 8:**
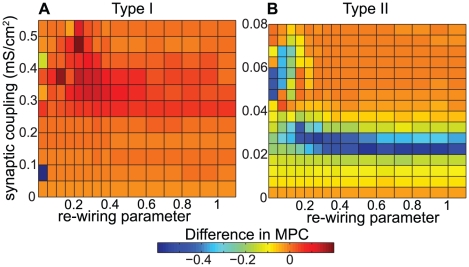
Differential effects of frequency modulation upon phase locking in cortical pyramidal neuronal networks. (A,B) Differences in MPC between high- and low-frequency networks as a function of network re-wiring and synaptic weight for (A) Type I and (B) Type II networks composed of cortical pyramidal cells. Values of 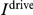 were the same as in Fig. 6.

Finally, Fig. 8 also supports the previously-presented trends. The differences in MPC between high- and low-frequency Type I networks were almost all very close to zero, while in the Type II networks there was a very significant region in which the MPC differences were negative. This negative region did not occupy as large an area in parameter space as it did for the bursting parameter, but that was due to the fact that the MPC saturated much more quickly than did the bursting parameter.

#### Cortical pyramidal neuron network driven by stochastic input currents

**Figure 9:**
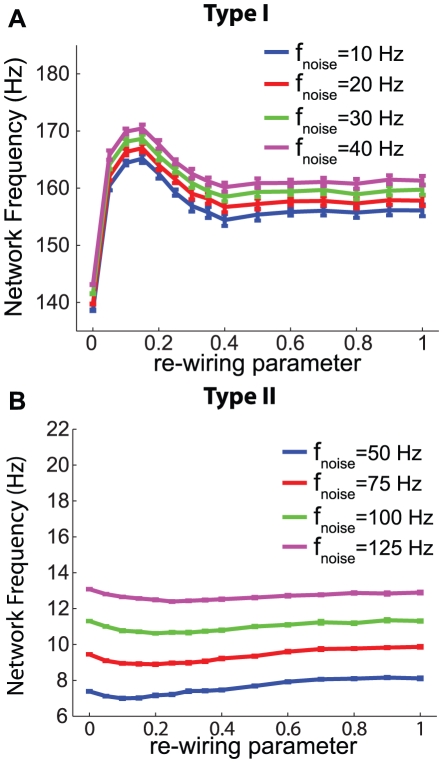
Average network frequency was directly modulated by noise frequency in stochastic input simulations. (A,B) Average network frequency as a function of the re-wiring parameter for various values of 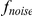 in (A) Type I and (B) Type II stochastic-input networks. Synaptic weight was set to 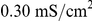 in (A) and 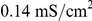 in (B).

**Figure 10:**
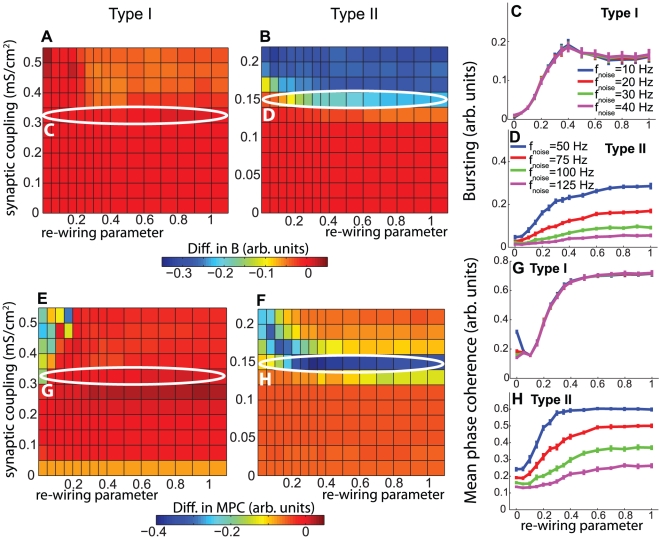
Differential effects of frequency modulation upon synchronization in stochastically-driven cortical pyramidal cell networks. (A,B) Differences in bursting parameter *B* between high- and low-frequency networks as a function of synaptic weight 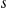 and the re-wiring parameter for (A) Type I and (B) Type II networks. For Type I networks, 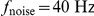 and 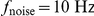 corresponded to high- and low-frequency networks, respectively, while for Type II networks, 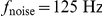 and 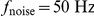 corresponded to high- and low-frequency networks, respectively. (C,D) Values of the bursting parameter as a function of the re-wiring parameter for four different values of 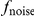, with synaptic coupling fixed at 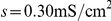 in (C) and 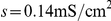 in (D). The circled regions in plots (A) and (B) were constructed by taking the difference between the highest- and lowest-frequency data points in (C) and (D). (E,F) Differences in MPC between high- and low-frequency networks as a function of synaptic weight and the re-wiring parameter for Type I and Type II networks. (G,H) Values of the MPC for four different values of 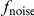, with synaptic coupling fixed at 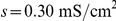 in (G) and 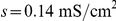 in (H). The circled regions in plots (E) and (F) were constructed by taking the difference between the highest- and lowest-frequency data points in (G) and (H). Line colors in plots (G) and (H) correspond to the same legend as in plots (C) and (D), respectively.

After we demonstrated the distinct synchronization response properties of Type I and Type II networks stimulated by varying levels of constant current, we next investigated the more biologically relevant context of stochastic stimulation. Here random current pulses were used to simulate neuronal drive coming from other brain modalities. Fig. 9 shows, as we would expect, that average network firing frequency consistently increased with 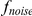, the average frequency at which sub-threshold current pulses were stochastically applied (see Methods for a more detailed description of this process), but remained largely independent of the network re-wiring parameter. The synchronization responses of the networks to frequency modulation were very similar to those described previously. The differences in bursting measure *B* between high- and low-frequency Type I networks were again very small for virtually all values of the network re-wiring parameter and coupling strength (Fig. 10A). The Type II networks, on the other hand, transitioned to synchrony at approximately 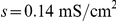 for almost all values of the re-wiring parameter (data not shown), at which point the differences in *B* became very negative (Fig. 10B), indicating once again that Type II networks were very sensitive to frequency modulation and that they had greater propensity for synchronization at low frequencies. Fig. 10C,D further illustrate the effect of increased frequency upon network synchrony for a particular value of the synaptic coupling.

Phase locking again largely followed the same trend as bursting, with the difference in MPC assuming values near zero for most of parameter space in Type I networks (Fig. 10E). The few very negative values seen, for high coupling and low re-wiring values, were most likely due to wave-propagation effects. Fig. 10F shows that Type II networks underwent a transition in MPC at 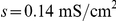, the same value at which the bursting values transitioned. Line plots for the MPC at this synaptic coupling value clearly demonstrate the increased propensity for synchronization at low frequency for Type II networks (Fig. 10H), while Fig. 10G shows the insignificant effect of frequency modulation upon Type I MPC.

## Discussion

We have shown that excitatory networks composed of neurons with either Type I or Type II PRC properties respond very differently to frequency modulation near firing threshold, with Type I network synchrony remaining largely unaffected by frequency modulation and Type II networks synchronizing much better at lower frequencies. This result is robust in virtually all network parameter regimes in which the network is capable of attaining any appreciable level of synchronization. While both Type I and Type II PRCs are modified by changes in frequency, only Type II PRCs change in qualitative profile. Specifically, the phase delay region, which is known to be critical in promoting synchrony, is severely attenuated. Increased frequency therefore tends to have little effect upon Type I networks, since there is no change in the PRC's contribution to synchrony, while in Type II networks it leads to depressed synchrony via the diminished phase delay region of the PRC. It should be noted that our simulations agreed with a large body of previous work showing that neurons with Type II membrane dynamics (as defined by the frequency-current curve) tend to synchronize better than neurons with Type I membrane dynamics, when coupled with excitation. Previous theoretical work indicates that when excitatory networks are driven with constant current, those composed of Type I neurons will not synchronize as well as those composed of Type II neurons [10], , a phenomenon which we observed in our simulations, since much larger synaptic coupling values were required in Type I networks to evoke levels of synchrony equivalent to those in Type II networks (Fig. 6). Previous theoretical [29] and experimental [30] work has also shown that neurons with Type I membrane dynamics respond to excitatory noisy input with much higher spike-time variability than do neurons with Type II membrane dynamics. This accords with the results of our simulations of networks stimulated by noisy current pulses, where again we saw that greater synaptic coupling was needed for Type I networks to synchronize as well as Type II networks (Fig. 10).

In this study, we focused on the implications for network synchronization of the observed frequency-dependence of PRCs. Our results suggest that the severe attenuation of the phase-delay region of Type II PRCs at increased firing frequencies contributes to the observed decline in network synchronization at such frequencies. Frequency-dependent modification of PRCs has been investigated before in complex, multi-compartment neuronal models [31], [32], but such results rely on dendritic effects and hence do not apply to our results using single-compartment neurons. It has been shown in a simple 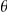-neuron model that low-threshold adaptation currents can produce negative regions in the PRC at low frequency [18], an effect which is probably due to the change in bifurcation structure induced by such currents [12]. From this perspective, the delay region of the PRC develops only at low frequency because the adaptation current is saturated at high frequency, resulting in its responding to excitatory stimulation with relatively smaller transient increases. Our work extends this insight by explaining the emergence and attenuation of delay regions in the PRCs of Morris-Lecar neurons, which have no adapting current. Our explanation applies to the cortical pyramidal model neuron, which does feature an adapting current, as well: it is the speed of low-threshold, hyperpolarizing currents relative to the interspike interval which determines the depth of the PRC delay region in Type II cells. For a fixed level of external current, the faster we made the 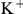 current in the Morris-Lecar neuron and the adapting 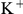 current in the cortical pyramidal neuron, the larger their PRC delay depths grew. It is therefore not only the saturation level of low-threshold, hyperpolarizing currents that is important, but also the speed with which they can respond to brief stimulation. In addition, our simulations showed that the PRC delay depth is not exclusively controlled by the effects of hyperpolarizing currents, but can be greatly affected by depolarizing currents as well. The faster we made the deactivation of the sodium current, the larger the delay depth grew, underscoring once again the importance of the speed of intracellular currents relative to the interspike interval.

The frequency-dependent synchronization which we have described in this paper could potentially be involved in any cognitive process, functional or pathological, which involves spatiotemporal pattern formation of neuronal populations. For example, cholinergically-induced switching between sensitivity and insensitivity to frequency modulation could be important in proper memory consolidation during slow wave and REM sleep, two states that are characterized by differing levels of acetylcholine in cortical and hippocampal regions. Frequency-mediated synchrony could also play a part in the binding of signals from multiple sensory modalities. Gamma oscillations (20–80 Hz) in cortical networks are believed to be generated by synchronous activity of fast-spiking interneurons [33], which generally exhibit Type II frequency-current relations and PRC profiles [27], [30]. While excitatory and inhibitory synaptic connections and gap junctions may participate in the synchronous firing of interneuron networks [34]–[37], our results suggest the importance of the cellular properties of the fast-spiking interneurons in generating synchrony. Additionally, the frequency-dependence of synchronization may provide a means to restrict synchronization to specific frequency bands. Finally, frequency modulation could contribute to the onset of epileptiform activity, and our results might help to explain recent evidence that synchrony decreases during seizures [38], [39].

At the same time, the importance of our results is not confined to these examples alone. Our findings point to the possibility that Type I and Type II excitatory networks function in two separate coding regimes, with Type I networks functioning in the rate coding regime and Type II networks functioning in the temporal coding regime, effectively acting as low-pass filters. Further experimental investigation into the interplay between cellular properties, frequency, and network synchronization is clearly required.
